# Regulation of the HTRA2 Protease Activity by an Inhibitory Antibody-Derived Peptide Ligand and the Influence on HTRA2-Specific Protein Interaction Networks in Retinal Tissues

**DOI:** 10.3390/biomedicines9081013

**Published:** 2021-08-13

**Authors:** Carsten Schmelter, Kristian Nzogang Fomo, Natarajan Perumal, Norbert Pfeiffer, Franz H. Grus

**Affiliations:** Department of Experimental and Translational Ophthalmology, University Medical Center, Johannes Gutenberg University, 55131 Mainz, Germany; cschmelter@eye-research.org (C.S.); kristianfomo@yahoo.de (K.N.F.); nperumal@eye-research.org (N.P.); norbert.pfeiffer@unimedizin-mainz.de (N.P.)

**Keywords:** HTRA2, neuroprotection, retina, interaction partners, co-immunoprecipitation, mass spectrometry

## Abstract

The mitochondrial serine protease HTRA2 has many versatile biological functions ranging from being an important regulator of apoptosis to being an essential component for neuronal cell survival and mitochondrial homeostasis. Loss of HTRA2 protease function is known to cause neurodegeneration, whereas overactivation of its proteolytic function is associated with cell death and inflammation. In accordance with this, our group verified in a recent study that the synthetic peptide ASGYTFTNYGLSWVR, encoding the hypervariable sequence part of an antibody, showed a high affinity for the target protein HTRA2 and triggered neuroprotection in an in vitro organ culture model for glaucoma. To unravel this neuroprotective mechanism, the present study showed for the first time that the synthetic CDR1 peptide significantly (*p* < 0.01) inhibited the proteolytic activity of HTRA2 up to 50% using a specific protease function assay. Furthermore, using state-of-the-art co-immunoprecipitation technologies in combination with high-resolution MS, we identified 50 significant protein interaction partners of HTRA2 in the retina of house swine (*p* < 0.01; log_2_ fold change > 1.5). Interestingly, 72% of the HTRA2-specific interactions (23 of 31 binders) were inhibited by additional treatment with UCF-101 (HTRA2 protease inhibitor) or the synthetic CDR peptide. On the other hand, the remaining 19 binders of HTRA2 were exclusively identified in the UCF101 and/or CDR group. However, many of the interactors were involved in the ER to Golgi anterograde transport (e.g., AP3D1), aggrephagy (e.g., PSMC1), and the pyruvate metabolism/citric acid cycle (e.g., SHMT2), and illustrated the complex protein interaction networks of HTRA2 in neurological tissues. In conclusion, the present study provides, for the first time, a comprehensive protein catalogue of HTRA2-specific interaction partners in the retina, and will serve as reference map in the future for studies focusing on HTRA2-mediated neurodegeneration.

## 1. Introduction

High temperature requirement protease A2 (HTRA2) is a mitochondrial protein belonging to the serine proteases of the HTRA family, distributed across all eukaryotic and prokaryotic species [1]. The mature HTRA2 protein contains a central serine protease domain and a C-terminal PDZ domain that packs against the protease active site and inhibits the proteolytic activity [2]. Some studies suggest that the proteolytic activity of HTRA2 is mainly regulated by protein−protein interactions, e.g., through the recognition of peptides from the C-terminal end of various proteins [3]. Moreover, Martins et al. (2003) [1] demonstrated that the proteolytic activity of the HTRA2 can be greatly enhanced by the disruption of the PDZ/protease interaction through the engagement of HTRA2-PDZ with a specific peptide ligands. Moreover, HTRA2 also comprises other domains and binding motifs such as the mitochondrial N-terminal localization signal (MLS), the transmembrane segment (TM), and the inhibitor of apoptosis (IAP)-binding motif (IBM) [4]. The serine protease HTRA2 is localized in the intermembrane space of the mitochondria where it acts as chaperone molecule by monitoring and controlling protein folding [5,6]. Thereby, the immature form of HTRA2 is anchored to the inner mitochondrial membrane by the TM motif and is released as an active 36-kDa HTRA2 protein fragment by autocatalytic processing [7]. Particularly, an apoptotic stimulus results in the translocation of HTRA2 from the mitochondria to the cytosol, and triggers, among others, cytochrome C-mediated caspase activation by the direct degradation of IAPs or via neutralization with the IBM binding motif [5,8,9]. Nevertheless, it also has been reported that transgenic *htra2^mnd^*^2^ mice deficient in HTRA2 protease activity (S276C missense mutation) show an obviously neurodegenerative phenotype accompanied by striatal neuron loss, severe muscle wasting, weight loss, and an early lethality [10,11]. In addition, the loss of HTRA2 protease activity increased the susceptibility of neuronal mitochondria for stress and also increased the sensitivity of *mnd2* mouse embryonic fibroblasts (MEF) for stress-induced cell death [10]. These findings, on the other hand, highlight the importance of proper HTRA2 protease function for neuronal cell survival and mitochondrial homeostasis. Recently, the serine protease HTRA2 was also identified as important novel regulator of autophagy [12] and was also associated with autophagic deficiency in the livers of aged rats [13]. Thereby, it induced autophagy through the digestion of HAX-1, a protein of the Bcl-2 family, that represses autophagy in a Beclin-1-dependent manner [12]. Interestingly, the HTRA2 protein activity was found be increased in the brain tissues of Alzheimer’s disease (AD) patients, and is supposed to promote neuroprotection by enhancing autophagic processes [14,15]. Additionally, it was already proven that an increased activity of HTRA2 promotes the degradation of mutant proteins (e.g., A53T α-synuclein) by autophagy [12], and might be also an important mechanism for amyloid plague removal in AD.

However, in a recent study of our group we identified the protein HTRA2 as a high-affinity interaction partner of the synthetic peptide ASGYTFTNYGLSWVR [16]. Thereby, this synthetic peptide encodes a complementary-determining region 1 (CDR1) of polyclonal antibody molecules, which was originally identified as a potential biomarker candidate in primary-open angle glaucoma (POAG) patients [17]. Furthermore, the synthetic CDR1 peptide ASGYTFTNYGLSWVR induced neuroprotective effects on retinal ganglion cells (RGCs) in an organ culture model for glaucoma, and was also accompanied by specific proteomic changes in the CDR1-treated retinal explants [16]. These important findings lead to the assumption that HTRA2 might represent a key player in neurodegenerative diseases such as glaucoma, and may serve as a potential therapeutic target in the future. To address this key question, the first aim was to evaluate how exactly the synthetic CDR1 peptide influences the proteolytic activity of HTRA2. Based on this, the main goal of the present study was to identify the direct protein interaction partners of HTRA2 in the retina of house swine (*Sus scrofa*) and to evaluate the influence of the synthetic CDR1 peptide on the HTRA2-specific retinal interactome.

## 2. Material and Methods

### 2.1. Synthetic Peptides

The synthetic peptides were manufactured in cooperation with the company Synpeptide Co., Ltd. (Shanghai, China), with a purity of 90%. The peptides were synthesized as follows: GQYYFV (termed PDZ-Opt), GGIRRV (termed PDZ-Sub), ASGYTFTNYGLSWVR (termed CDR), and YVWAGSTLSRTGNFY (termed scrambled CDR). The synthetic peptides for the mutational analysis were purchased from G&W Biotechnologie GmbH (Mainz, Germany) with a purity of 90%. The peptides were synthesized as follows: ASGATFTNYGLSWVR, ASGYAFTNYGLSWVR, and ASGYTATNYGLSWVR. All of the peptides were synthesized without any modification.

### 2.2. HTRA2 Protease Activity Assay

The HTRA2 protease activity assay was performed as described by Martins et al. (2003) [1], but with some slight modifications. The experiment was performed with 100 nM recombinant HTRA2 protein (Novoprotein Scientific INC., Summit, NJ, USA) in a protease assay buffer (50 mM tris(hydroxymethyl)-aminomethane (TRIS), 0.5 mM ethylenediaminetetraacetic acid (EDTA), and 1 mM dithiothreitol (DTT), pH 8.0) containing 10 µM H2-optimal substrate (Innovagen AB, Lund, Sweden). The H2-optimal substrate is commonly used to measure the protease activity of HTRA2 and consists of a structural quencher and a fluorophore unit. The cleavage of the substrate leads to the disruption of the quencher/fluorophore complex and the degradation rate can be measured by fluorescence. The different sample groups were treated with 50 µM PDZ-Opt, 50 µM PDZ-Sub, 50 µM synthetic CDR1 peptide, or 30 µM UCF-101, respectively. The drug compound UCF-101 is an inhibitor of the catalytic domain of HTRA2 and was purchased from Sigma Aldrich (Darmstadt, Germany). The concentrations of the peptide ligands for the HTRA2 protease activity assay were chosen from the previous publication [1]. The concentration of UCF-101 was determined to be optimal from the preliminary experiments. In addition, a positive control containing only 100 nM recombinant HTRA2 and a negative control (only protease assay buffer) were included in this experiment. All of the sample groups were incubated for 1 h at 37 °C to determine the maximum degradation rate of HTRA2. For the analysis of the enzymatic reaction rate of HTRA2, all samples were measured subsequently after the addition of the 10 µM H2-optimal substrate at RT. Fluorescence was monitored on a Spark^®^ 10 M multimode microplate-reader (Tecan Trading AG, Männedorf, Switzerland) with an excitation at 320 nm and a fluorescence emission at 405 nm. The fluorescence background of the substrate was substracted from the fluorescence intensities of the different the sample groups to normalize the data.

### 2.3. Retina Extraction and Homogenization

The retina extraction and homogenization were performed as already described in previous publications [16,18]. The use of animal by-products of house swine for scientific research purposes was approved by the Kreisverwaltung Mainz-Bingen in Germany (Identification Code: DE 07 315 0006 21, approved on 13 January 2014). In brief, retinal tissues were extracted from freshly removed porcine eye bulbs (house swine, *Sus scrofa domestica* Linnaeus, *n* = 30) provided by the local slaughterhouses (Landmetzgerei Harth, Stadecken-Elsheim, Germany). Afterwards, the retinal tissues were homogenized in 2 mL screw cap microtubes filled with 1.4/2.8 mm ceramic beads and 1 mL Tissue Protein Extraction Reagent (T-PER, Thermo Fisher Scientific, Rockford, IL, USA) using a Percellys^®^ 24 homogenizer (VWR International GmbH, Darmstadt, Germany) [19]. The homogenized retinal samples were then centrifuged at 14,000× *g* for 15 min at 4 °C. The protein-containing supernatant was exchanged in a 100 µL phosphate-buffered saline (PBS) using a 3 kDa Amicon^®^ filtration unit (Millipore, Billerica, MA, USA) and, prior to this, was extensively washed in order to completely remove traces of the T-PER buffer. Afterwards, the samples were pooled and the protein concentration was determined using a Pierce Bicinchoninic Acid (BCA) Protein Assay Kit (Thermo Fisher Scientific, Rockford, IL, USA) according to the manufacturer’s protocol, which provided 5 mg aliquots for further analysis.

### 2.4. Co-Immunoprecipitation (Co-IP) of HTRA2 Protein Interaction Partners

The recombinant protein HTRA2 with a C-terminal 6xHis-tag motif (Novoprotein Scientific INC., Summit, NJ, USA) and 40 μL HisPurTM Ni-NTA magnetic beads (Thermo Fisher Scientific, Rockford, IL, USA) were spiked into 5 mg homogenized porcine retina. The experiment comprised of four groups: control homogenate (CTRL group), homogenate with 2 μg recombinant HTRA2 (HTRA2 group), homogenate with 2 μg recombinant HTRA2 and 10 μg UCF-101 (UCF-101 group), and homogenate with 2 μg recombinant HTRA2 and 10 μg synthetic CDR peptide (CDR group). The Co-IP of the recombinant HTRA2 and the enrichment of the respective interaction partners was performed with three independent biological replicates for each group (CTRL, HTRA2, UCF-101, and CDR group). After incubation at 4 °C overnight, the 6xHis-tagged protein HTRA2 with the bounded protein interaction partners (co-immunoprecipitation) was isolated with the previously added HisPurTM Ni-NTA magnetic beads (Thermo Fisher Scientific, Rockford, IL, USA) using a magnetic stand. Each bead group was washed twice with 400 μL PBS and the bounded HTRA2 including the protein interaction partners were eluted by pH shift. The protein concentration of each eluate fraction was determined as described above. Afterwards, 50 μg of each eluate fraction was evaporated using a speed vacuum concentrator (SpeedVac) (Eppendorf, Hamburg, Germany) for 45 min at 30 °C and was stored afterwards at −20 °C.

### 2.5. 1D SDS-PAGE and In-Gel Trypsin Digestion

The protein eluate fractions of each group (50 μg; *n* = 3 biological replicates per group) were separated by 1D SDS PAGE on 10-well NuPAGE^®^ 12% Bis-Tris gels using NuPAGE™ MOPS SDS Running Buffer 20X (Thermo Fisher Scientific, Rockford, IL, USA). The 1D SDS PAGE was performed with the XCell *SureLock*^TM^ Mini-Cell Electrophoresis System (Thermo Fisher Scientific, Rockford, IL, USA) under denatured and reduced conditions, as already described in previous publications [16,20,21,22]. The protein separation was performed for 2 h at 4 °C using 150 V, and then the gels were stained with the Novex Colloidal Blue Staining Kit (Thermo Fisher Scientific, Rockford, IL, USA) according to the manufacturer’s protocol. The next day, the gels were destained overnight and scanned using a DGP-9042 scanner (Brother Industries, Nagoya, Japan) at 600 dpi. In-gel trypsin digestion was performed as already described in previous publications [16,20,21,22,23]. In brief, each protein lane was divided into 17 slices and was subsequently cut into small pieces. The gel pieces were first destained with 100 mM ammonium bicarbonate (NH_4_HCO_3_)/acetonitrile (ACN) (1:1, *vol*/*vol*) and then dehydrated with pure ACN. Afterwards, disulfide bond reduction was applied with 10 mM DTT in 100 mM NH_4_HCO_3_ for 30 min at 56 °C. Subsequently, the alkylation step was performed with 55 mM iodoacetamide (IAA) in 100 mM NH_4_HCO_3_ for 30 min at RT in the dark. Accordingly, the samples were dehydrated once again with pure ACN prior to digestion with trypsin (Promega, Madison, WI, USA) overnight at 37 °C. The concentration of the trypsin solution was 13 ng/μL dissolved in 100 mM NH_4_HCO_3_ 10% ACN. The next day, the supernatant was collected in new reaction tubes and the protein digest was extracted from the remaining gel pieces using 100 μL of extraction buffer (10% formic acid (FA) in 70% can) for 30 min at 37 °C. Both of the supernatant fractions were pooled, dried in a SpeedVac at 30 °C, and then subsequently stored at −20 °C prior to further analysis. Afterwards, the protein digest was purified with the SOLAµ™ HRP SPE spin plates (Thermo Fisher Scientific, Rockford, IL, USA) according to the manufacturer’s protocol. The purified peptides were evaporated and concentrated in the SpeedVac at 30 °C.

### 2.6. LC-MS/MS Analysis

The liquid-chromatography-mass spectrometry (LC-MS) analysis of the samples was performed with the Hybrid Linear Ion Trap-Orbitrap MS system (LTQ Orbitrap XL; Thermo Fisher Scientific, Rockford, IL, USA), which is a well-established and commonly used system in our laboratory [16,20,21,22,23]. The MS system was coupled to a Rheos Allegro pump (Thermo Fisher Scientific, Rockford, IL, USA) downscaled to a capillary flow rate (6.7 ± 0.3 μL/min). The reverse phase chromatography prior to MS analysis was performed using a Jupiter^®^ 5 µm C18 300 Å (150 × 0.5 mm) analytical column system (Phenomenex, Aschaffenburg, Germany). All of the samples were dissolved in 10 μL of 0.1% trifluoroacetic acid (TFA) and 6 µL of each sample was injected into the system for the LC-MS analysis. Solvent A consisted of 0.1% FA in water and solvent B consisted of 0.1% FA in ACN. The gradient for the peptide elution was set to 60 min, as follows: 15–40% B (0–30 min), 40–60% B (30–35 min), 60–90 B (35–45 min), and 90–10% B (45–60 min). LTQ-Orbitrap was operated in the positive ionization mode and in the data dependent acquisition (DDA) mode. The full MS spectra scan (from *m*/*z* 300 to 2000) was recorded in the orbitrap with a resolution of 30,000 to *m*/*z* 400 and the automatic gain control (AGC) was set to 1 × 10^6^ ions. For the lock mass option, polydimethylcyclosiloxane *m*/*z* 445.120025 was used for the internal calibration. The dynamic exclusion mode was applied as follows: repeat count = 3, repeat duration = 600 s, exclusion size list = 500, exclusion time = 600 s, and the exclusion mass width ± 10 ppm. The five most intense precursor ions were selected for fragmentation in the ion trap with collision induced decay (CID) fragmentation using normalized collision energy of 35%. The mass spectrometry proteomics data were deposited to the ProteomeXchange Consortium via the PRIDE [24] partner repository with the dataset identifier PXD024442.

### 2.7. Protein Identification and Quantification

The MS output data were processed using the bioinformatics software MaxQuant v. 1.6.1.0 (Max Planck Institute for Biochemistry, Martinsried, Germany). Database searches were performed using the implemented Andromeda search engine to match the MS/MS spectra with the updated SwissProt database [25]. The tandem MS spectra were searched against the SwissProt databases with the taxonomies *Homo sapiens* (date: 14/02/2020, number of sequences: 20,364) and *Sus scrofa* (date: 14/02/2020, number of sequences: 1433) using the following search parameters: peptide ion mass tolerance of ±30 ppm, fragment ion mass tolerance of 0.5 Da, tryptic cleavage, maximum of two missed cleavage sites, carbamidomethylation as the fixed modification, and acetylation (N-terminal protein) and oxidation as the variable modifications. Because of the limited access to proper public proteomic databases of the house swine (*Sus scrofa*) [26], we included the species-related proteomic database of *Homo sapiens* to increase the protein identification rates. Only reviewed database entries were used for protein identification and quantification. Moreover, all of the identified proteins were filtered with a false discovery rate (FDR) < 1%.

### 2.8. Data Analysis and Statistics

The statistical analysis of the MS data was processed using Perseus software version 1.6.6.0 (Max Planck Institute of Biochemistry, Martinsried). Protein identifications were filtered for contaminants, reversed hits, and “only identified by site”. For the identification of the significant protein interaction partner of HTRA2, we used the MaxQuant-specific iBAQ (Intensity Based Absolute Quantification) values of the proteins for the statistical quantitative analysis. The IBAQ value was obtained by dividing the raw intensity of each protein by the number of theoretical peptides, and was already used for label-free immunoprecipitation experiments [27]. At first, the intensities of all detected proteins were log_2_ transformed. In order to identify only the true interaction partners of HTRA2, the quantitative data of each group (HTRA2, UCF-101, and CDR groups) were normalized to the control bead group (CTRL). All of the true protein hits needed to be detected in at least three biological replicates of the CTRL group or in least three biological replicates of the other experimental groups (HTRA2, UCF-101, or CDR group). Missing protein intensity values were imput on the basis of the normal distribution of the data (width: 0.3, down shift: 1.8). For the identification of the HTRA2-specific interaction partners, only missing values of the CTRL group were imput in accordance with previous publications [16,28]. Volcano plot analysis with the following filtering criteria (*p* < 0.01; log_2_ fold change > 1.5) revealed significantly enriched protein interaction partners of HTRA2 in all three experimental groups (HTRA2, UCF-101, and CDR group). Further statistical analyses and graphical presentation of the data were performed by using Statistica version 13 (Statsoft, Tulsa, OK, USA) or EXCEL 2016 functions.

### 2.9. Pathway and Enrichment Analysis

Gene Ontology enrichment analysis was applied using the functional annotation tool DAVID (http://david.abcc.ncifcrf.gov/home.jsp; accessed date: 1 June 2020) [29]. The GO terms with *p*-values < 0.05 were considered to be significantly enriched. The enrichment analysis was performed with taxonomy *Homo sapiens* as the reference database. In addition, a further pathway and enrichment analysis was performed with the gene annotation software Metascape (http://metascape.org; accessed date: 1 June 2020). Furthermore, the Metascape-specific molecular complex detection (MCODE) algorithm was used to detect densely connected network components (protein interaction networks) [30].

### 2.10. Targeted MS Analysis

In order to validate the protein interaction partners of HTRA2 revealed in the discovery study, we performed a targeted MS strategy via accurate inclusion mass screening (AIMS) technology [31]. Thereby, the peptides of interest were listed in the software inclusion list and the LTQ Orbitrap XL MS system only required MS/MS scans of the parent ions if the listed peptide was detected with an accurate mass and charge state. Selection of the peptides for MS/MS fragmentation was carried out manually using the unique peptides of the HTRA2 specific interaction partners revealed from the discovery study. The selected peptides needed to be fully tryptic with a maximum of one missed cleavage, as described earlier [17]. For this experiment, we repeated the Co-IP of the HTRA2-specific protein interaction partners ± treatments (see method Section 2.4) and subjected the respective eluate fractions to further in-solution trypsin digestion, as described elsewhere [17]. In this case, the LTQ Orbitrap XL MS system was coupled online to an EASY-nLC 1200 system (Thermo Fisher Scientific, Rockford, IL, USA) using a PepMap C18 column system (75 µm × 500 mm; Thermo Fisher Scientific, Rockford, IL, USA). The dynamic exclusion mode was applied as follows: repeat count = 3, repeat duration = 30 s, exclusion size list = 100, exclusion time = 180 s, and the exclusion mass width ± 10 ppm. Protein identification and quantification were performed as described in Section 2.7.

## 3. Results

### 3.1. HTRA2 Protease Activity Assay

To determine the proteolytic activity of HTRA2 we used the fluorescent H2 optimal substrate developed by Martins et al. (2003) [1]. The optimal substrate H2 encoded a short specific amino acid sequence, which is preferentially cleaved by HTRA2, and was also modified with a fluorescent dye and quencher. Enzymatic cleavage of the H2 optimal substrate resulted in an increased release of the fluorescent dye, which was positively correlated with the proteolytic activity of HTRA2. Under physiological conditions, the proteolytic activity of HTRA2 is suppressed by the C-terminal PDZ domain, which blocks the access of potential substrates to the catalytic domain by steric hindrance. The binding of short hydrophobic polypeptides to the allosteric PDZ domain can induce specific conformational changes of HTRA2 and are able to regulate its proteolytic activity. Therefore, Martins et al. (2003) [1] specifically designed the peptide ligands PDZ-Opt (GQYYFV) and PDZ-Sub (GGIRRV), which are known to increase the proteolytic activity of HTRA2 in this assay.

**Figure 1:**
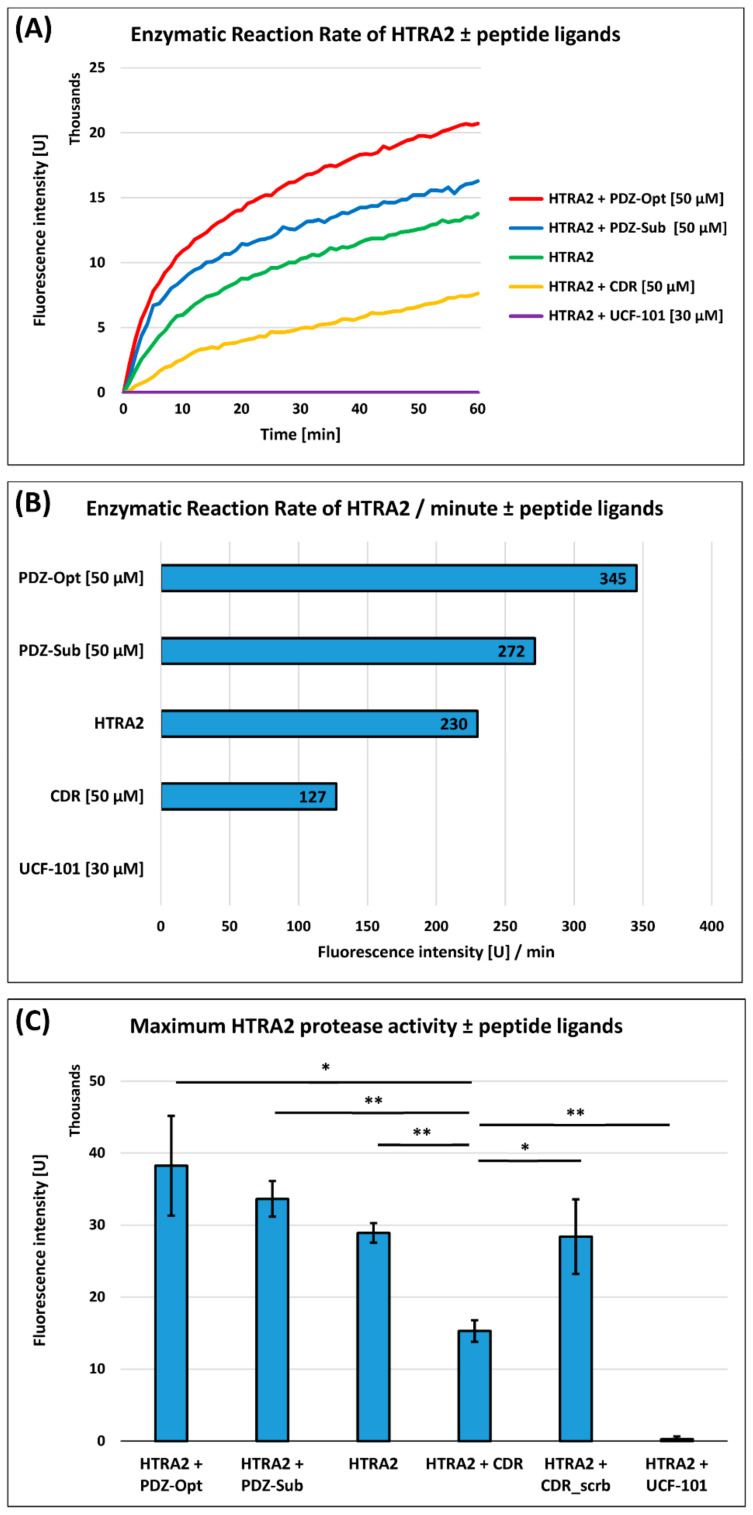
Determination of the HTRA2 protease activity using the fluorescent H2 optimal substrate. Enzymatic cleavage of the H2 optimal substrate elicited fluorescence emission at 405 nm, which is correlated with the proteolytic activity of HTRA2. (**A**) Enzymatic reaction rates of HTRA2 ± PDZ domain-binding peptide ligands PDZ-Opt (50 µM), PDZ-Sub (50 µM), and the synthetic CDR peptide (50 µM). In addition, HTRA2 was treated with the specific protease inhibitor UCF-101 (30 µM). (**B**) This graphic illustrates the enzymatic reaction rates of HTRA2 per time units (minutes). Average degradation rates (U/min) of the H2 optimum substrate are shown for the groups HTRA2 ± PDZ-Opt (50 µM), PDZ-Sub (50 µM), the synthetic CDR peptide (50 µM), and UCF-101 (30 µM). (**C**) Maximum degradation rates of the H2 optimum substrate by HTRA2 at 37 °C for 1 h. HTRA2 was treated with the PDZ domain-binding peptide ligands PDZ-Opt (50 µM), PDZ-Sub (50 µM), the synthetic CDR peptide (50 µM), and UCF-101 (30 µM) (*n* = 3 for all groups). In addition, HTRA2 was treated with a scrambled CDR peptide analog (50 µM; *n* = 3). **: *p* < 0.01, *: *p* < 0.05.

Using these two PDZ-binding peptide ligands as reference values, the enzymatic reaction rates of HTRA2 could be efficiently increased compared with the untreated positive control (only HTRA2; see Figure 1A). The average degradation rates of the optimal substrate H2 could be increased up to 18% by PDZ-Sub (272 U/min) and up to 50% by PDZ-Opt (345 U/min) with respect to the untreated positive control (229 U/min; see Figure 1B). On the other hand, the synthetic CDR peptide (ASGYTFTNYGLSWVR) clearly inhibited the proteolytic activity of HTRA2 (see Figure 1A,B) and decreased the average degradation rate of the optimal substrate H2 by around 45% (127 U/min) compared with the untreated positive control (229 U/min). In addition, the protease Inhibitor UCF-101 completely abolished the degradation rate of the optimal substrate H2 (0 U/min) by HTRA2.

In the next part of the analysis, we were interested in the maximum degradation rates of HTRA2 ± treatments (UCF-101, CDR, PDZ-Sub, and PDZ-Opt) by incubating all of the samples for 1 h at 37 °C (see Figure 1C). As already proven by the enzymatic reaction rate, the synthetic CDR peptide significantly decreased the maximum degradation rates of the H2 optimal substrate by HTRA2 compared with the other groups (*p* < 0.01 compared with PDZ-Sub, HTRA2, and UCF-101; *p* < 0.05 compared with PDZ-Opt; *n* = 3 for all of the groups). In addition, we treated a further sample group with a scrambled CDR peptide analog (*YVWAGSTLSRTGNFY*), which was unable to induce the suppression of the HTRA2 protease activity (*p* < 0.05 compared with CDR, *n* = 3). These finding confirmed the importance of the sequence specificity of the CDR peptide for its inhibitory effect on HTRA2.

Furthermore, by using PDZ-binding peptide libraries, Martins et al. (2003) [1] demonstrated the preferred presence of hydrophobic amino acids Y or F at positions −1, −2, and −3 for proper binding to the PDZ domain, which were also considered for the design of the peptide ligand PDZ-Opt (GQ**YYF**V). Therefore, this sequence part was identified as a major binding motif to the PDZ domain and can be also found as crucial part in the synthetic CDR peptide (ASG**YTF**TNYGLSWVR).

Targeted alanine substitution (mutational analysis) of the potential binding motif in the synthetic CDR revealed the particular important presence of Y(4) and F(6) for the inhibitory effect on HTRA2 (see Figure S1). Interestingly, the targeted substitution of T(5) to A(5) in the potential binding motif significantly strengthened the inhibitory effect of the synthetic CDR peptide towards HTRA2.

### 3.2. Identification of HTRA2-Specific Protein Interaction Partners in Retinal Tissues

**Figure 2:**
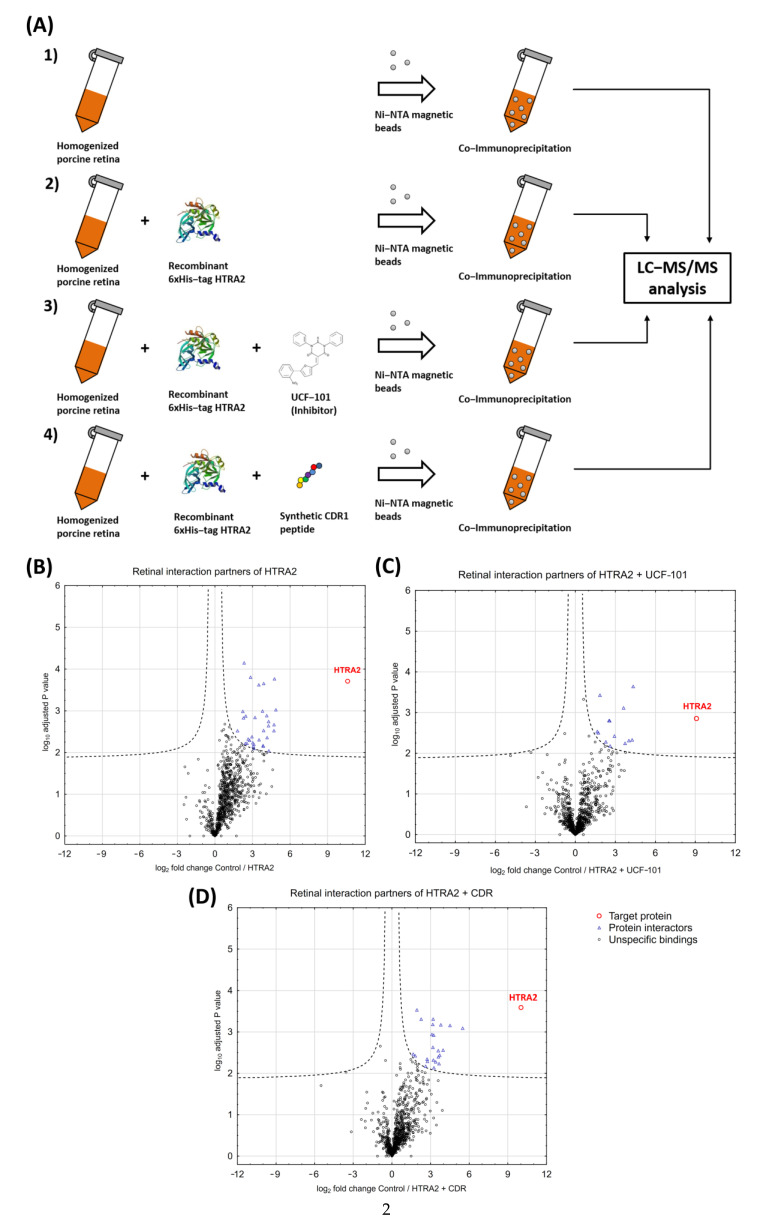
Experimental workflow showing the co-immunoprecipitation of HTRA2-specific protein interaction partners (**A**) from the retinal tissues of house swine (*Sus scrofa*) and (**B**–**D**) the results of the mass spectrometric data analysis. (**A**) Recombinant HTRA2 (2 µg) with an N-terminal 6xHis-tag was spiked into 5 mg homogenized porcine retina lysate ± 10 µg UCF-101 or 10 µg synthetic CDR peptide (*n* = 3 for all of the experimental groups). A control bead group was also included in this experiment to distinguish the HTRA2-specific protein interactors from unspecific binders (*n* = 3). Ni-NTA magnetic beads (40 µL) were added to each sample to enrich recombinant 6xHis-tagged HTRA2 with the respective protein interaction partners. After incubation, all of the bead fractions were extensively washed, and the remaining attached proteins were eluted by pH shift. All of the eluate fractions were further subjected to in-gel trypsin digestion and measured by LC-MS/MS. (**B**) Volcano plot showing the log_2_ fold change plotted against -log10-adjusted *p* values from samples with recombinant 6xHis-tag HTRA2 versus samples from the control bead group (*p* < 0.01; log_2_ fold change > 1.5); 31 proteins were identified as significant interaction partners of HTRA2. (**C**) Volcano plot analysis revealed 13 significant interaction partners in samples with 6xHis-tag HTRA2 and 10 µg UCF-101 compared with the control bead group (*p* < 0.01; log_2_ fold change > 1.5). (**D**) Volcano plot identified 23 significant protein interactors in samples with 6xHis-tag HTRA2 and 10 µg synthetic CDR peptide compared with the control bead group (*p* < 0.01; log_2_ fold change > 1.5).

For the identification of HTRA2-specific protein interaction partners in the retinal tissues of house swine (*Sus scrofa*), we performed co-immunoprecipitation (Co-IP) experiments with recombinant 6xHis-tag HTRA2 followed by quantitative LC-MS-based proteomics (see Figure 2A and File S1). In brief, 2 µg recombinant 6xHis-tag HTRA2 (HTRA2 group) was spiked into 5 mg homogenized porcine retina and was subsequently enriched with the bounded protein interaction partners via Ni-NTA affinity purification (*n* = 3 biological replicates). In addition, two further sample groups were treated with 10 µg UCF-101 (UCF-101 group) or 10 µg synthetic CDR peptide (CDR group) to unravel the influence of both molecules on the HTRA2-specific protein interactome in the retinal tissues (*n* = 3 biological replicates for each group). Eluate fractions of each group were separated by 1-D SDS PAGE (Figure S2) and were subsequently subjected to in-gel trypsin digestion. To distinguish unspecific from HTRA2-specific binders, we also included a control Ni-NTA bead group (CTRL group) in the quantitative MS analysis (*n* = 3 biological replicates).

For the statistical analysis, the relative protein abundances of the HTRA2, UCF-101, and the CDR group were “normalized” to the protein abundances of the CTRL group, as shown by the Volcano plot analyses (Figure 2B–D and File S2). As first result, recombinant 6xHis-tag HTRA2 was successfully enriched in each group by affinity purification (see Figure 2B–D and Figure S2), and also no significant difference (*p* > 0.05) regarding HTRA2 protein recovery was observed. Furthermore, co-enriched protein species fulfilling the strict filtering criteria (*p* < 0.01; log_2_ fold change > 1.5) of the Volcano plot analyses were identified as significant interaction partners of HTRA2 (in total 50 proteins in all three groups; see Table 1). In summary, 31 significant protein interaction partners were identified in the HTRA2 group, 13 significant interactors were enriched in the UCF-101 group, and 23 significant binders were revealed in the CDR group.

**Figure 3:**
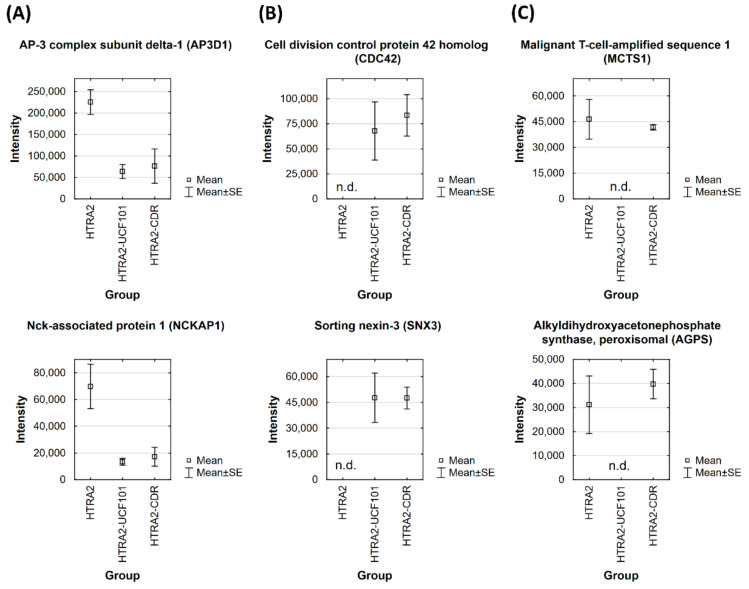
Bar plots of exemplary protein interaction partners enriched with recombinant 6xHis-tag HTRA2 ± 10 µg UCF-101 or 10 µg synthetic CDR peptide. (**A**) Bar plots on the left show significant HTRA2-specific interaction partners AP3D1 and NCKAP1, which were diminished by additional treatment with UCF-101 or synthetic CDR peptides. (**B**) Bar plots in the middle represent protein interactors CDC42 and SNX3, which were specifically enriched in samples with 6xHis-tag HTRA2 ± UCF-101 or synthetic CDR peptides. (**C**) Bar plots on the right indicate the enrichment of HTRA2-specific protein interaction partners MCTS1 and AGPS, which were specifically suppressed by the treatment with UCF-101. All of the protein interactors of HTRA2 were significantly enriched compared with the control bead group (*p* < 0.01; log_2_ fold change > 1.5). Not detectable (n.d.) proteins were not present in at least three biological replicates of the respective group (HTRA2, UCF-101, or CDR group).

Interestingly, most of the significant interactions (23 of 31 binders) in the HTRA2 group were diminished in the UCF-101 or the CDR group (*p* > 0.01; log_2_ fold change < 1.5 compared to CTRL group). Exemplary bar plots of the affected protein interactors AP3D1 and NCKAP1 are shown in Figure 3A. On the other hand, about 54% of the significant binders (7 of 13 interactors) in the UCF-101 group were also found to be significantly enriched in the CDR group (see Table 1). Bar plots of the proteins CDC42 and SNX3 are indicated in Figure 3B, which were exclusively enriched in the UCF-101 and CDR group. In addition, six interaction partners were only significantly enriched in the HTRA2 and CDR group, but failed to pass to the filtering criteria in the UCF-101 group (*p* < 0.01; log_2_ fold change > 1.5 compared to the CTRL group). Representative bar plots of the interaction partners MCTS1 and AGPS are shown in Figure 3C. Moreover, 16% of all HTRA2-specific protein binders (e.g., SFRP1) were uniquely enriched in the CDR group, whereas around 8% were uniquely identified as significant interactors (e.g., COPE) in the UCF-101 group (see Table 1).

For further validation of the HTRA2-specific interaction partners, we performed a targeted MS analysis via accurate inclusion mass screening (AIMS) technology [31]. AIMS targets only selected peptides of interest and is much more sensitive than undirected discovery proteomics experiments. For validation, we repeated the HTRA2 Co-IP experiments ± treatments (UCF-101 and CDR), and subjected the eluate fractions to further in-solution trypsin digestion (*n* = 3 biological replicates per group; see Section 2.10). This time, the LC-MS system “only” recorded the unique peptide sequences of the previously identified HTRA2 interaction partners. AIMS analysis verified the increased co-enrichment of the target proteins FNLA, NCKAP1, POTEE, PSMC1, and SEC13 in the HTRA2 group (see Figure S3). The abundances of the target proteins were significantly diminished in the CDR group (*p* < 0.05) and showed at least a slight decrease in the UCF-101 group, even if this effect was not supported by statistical significance (*p* > 0.05).

### 3.3. GO Enrichment and Pathway Analysis of the HTRA2-Specific Retinal Interactome

**Figure 4:**
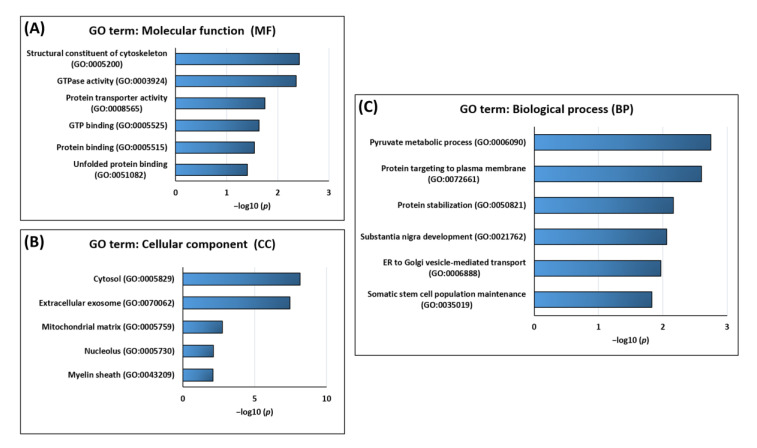
GO enrichment analysis of all HTRA2-specific protein interaction partners. Negative log10-adjusted *p*-values (*p* < 0.05) for the significantly enriched GO terms are shown for the molecular function (**A**), the cellular component (**B**), and the biological process (**C**) of all interactors.

In the next step of the analysis, a gene ontology (GO) enrichment analysis was performed using functional annotation tool DAVID to explore the versatile molecular functions, the cellular localization, and the induced biological processes of the HTRA2-specific protein interaction partners (see Figure 4 and File S3). The most significant molecular functions of the HTRA2-specific interactors were structural constituents of the cytoskeleton, GTPase activity, protein transporter activity, GTP binding, protein binding, and unfolded protein binding (see Figure 4A). Furthermore, the majority of the identified binders were located in the cytosol, extracellular exosomes, mitochondrial matrix, nucleolus, or myelin sheath (see Figure 4B). Highlighting the biological processes of the HTRA2-specific interactors, the most significant functions were pyruvate metabolic processes, protein targeting to plasma membrane, protein stabilization, *substania nigra* development, ER to Golgi vesicle-mediated transport, and somatic stem cell population maintenance (see Figure 4C).

**Figure 5:**
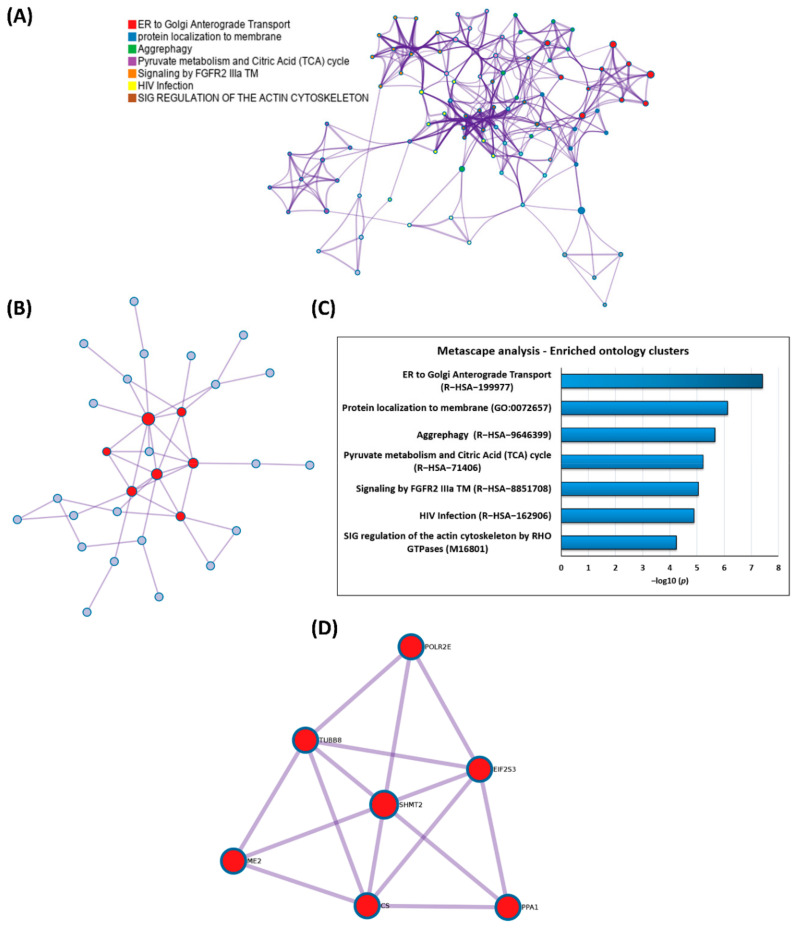
Metascape analysis of the identified protein interaction partners of HTRA2. (**A**) The seven most significant protein interaction pathways identified by enriched GO and Reactome terms colored by cluster. (**B**) The seven most significant protein interaction pathways with the corresponding −log_10_-adjusted *p* values. (**C**) Protein−protein interaction (PPI) networks with densely connected components (red) identified by the molecular complex detection (MCODE) algorithm. (**D**) Magnified view of the significant PPI network comprising the HTRA2-specific interactors POLR2E, TUBB8, SHMT2, EIF2S3, ME2, CS, and PPA1.

Furthermore, to provide deeper insights into the complex regulatory interaction networks of the HTRA2-specific protein binders, an additional functional annotation analysis via the Metascape platform was performed (see Figure 5 and File S3). The six most significant signaling pathways determined by enriched GO and Reactome terms are shown in Figure 5A,B. The majority of the HTRA2-specific interaction partners were involved in ER to Golgi anterograde transport, protein localization to membrane, aggrephagy, pyruvate metabolism and citric acid (TCA) cycle, signaling by FGFR2 IIIa TM, HIV infection, and SIG regulation of the actin cytoskeleton by RHO GTPases. The HTRA2-specific protein interaction networks are shown in Figure 5A and their corresponding −log_10_-adjusted *p* values are demonstrated in Figure 5B. In addition, the Metascape molecular complex detection (MODE) algorithm identified a significant protein interaction network of HTRA2-specific binders, which are known to interact with each other (see Figure 5C). The densely connected network components (red dots) are illustrated in Figure 5D and compromise the six HTRA2-specific interaction partners POLR2E, TUBB8, SHMT2, EIF2S3, ME, CS, and PPA1. The independent enrichment analysis of the MCODE components revealed the major signaling pathways aerobic respiration (GO: 0009060) and carbon metabolism (KEGG: hsa01200) for these six HTRA2-specific interactors (see File S3).

## 4. Discussion

The main focus of the present study is based on the target protein HTRA2 (high temperature requirement A2), which has been associated with many versatile functions so far, ranging from being an important inducer of apoptosis [8,9,32] to being a serious regulator of essential neuroprotective functions [10,11,33]. In general, HTRA2 acts as mitochondrial serine protease and promotes the degradation of misfolded or aggregated protein structures [5]. Thereby, specific substrate molecules (e.g., XIAP) are directly cleaved by the proteolytic function of HTRA2 [34], whereas other cytotoxic protein deposits (e.g., mutant A53T α-synuclein) are forwarded for degradation by HTRA2-induced autophagic processes [12].

However, in a recent study by our group, it was demonstrated that the synthetic CDR1 peptide ASGYTFTNYGLSWVR induced neuroprotection on retinal ganglion cells (RGC) in an in vitro glaucoma model and possessed a high affinity for the target protein HTRA2, verified by immunoprecipitation experiments [16]. In accordance, these neuroprotective effects were accompanied by specific proteomic changes in the retinal tissues showing a decreased expression of cellular stress response markers (e.g., HSP90AA1) and increased levels of neuroprotective and anti-oxidative proteins (e.g., TXN) [16]. By using an HTRA2-specific protease activity assay, developed by Martins et al. (2003) [1], it could finally be proven that the synthetic CDR peptide ASGYTFTNYGLSWVR significantly inhibited the proteolytic activity of HTRA2 compared with the untreated positive control (see Figure 1A–C). In contrast, the HTRA2 protease activity could be clearly increased by using the two reference peptides, PDZ-Opt (GQYYFV) and PDZ-Sub (GGIRRV), compared with the untreated positive control (see Figure 1A–C). The peptide ligand PDZ-Opt was specifically designed to maximize the proteolytic activity of HTRA2 via a strong interaction with the inhibitory PDZ domain [1,35]. The peptide ligand PDZ-Sub, in contrast, represents a peptide generated by HTRA2 auto-proteolysis, and was reported to activate the protease function less efficiently than PDZ-Opt [1], as proven in our experiments (see Figure 1A–C). However, a sequential comparison of the reference peptide PDZ-Opt with the inhibitory CDR revealed, in particular, the sequence motif ASG(**YTF**)TNYGLSWVR as a potential binding motif to the PDZ domain. Targeted mutational analysis of the potential binding motif in combination with the protease activity assay confirmed the importance of the hydrophobic amino acids Y(4) and F(6) for the inhibitory effect on HTRA2 (see Figure S1), which were also found to be highly presented in PDZ-binding peptide ligands, particularly at positions −1, −2, and −3 [1,2]. Remarkably, the inhibitory effect of the synthetic CDR was significantly increased by the mutational substitution of T(5). Alanine (A; side chain -CH_3_) has a shorter side chain compared with T (side chain: CH_3_-COOH), and might explain the enhanced inhibitory effect on HTRA2 at this specific position. In addition, a similar peptide ligand AGYTGFV, such as the assumed binding motif of the CDR, was proven to bind with a high affinity (Glide score: −7.903 kcal/mol) to the HTRA2-PDZ domain [35]. Based on these findings, it can be assumed that the synthetic CDR peptide might regulate the proteolytic activity of HTRA2 via engagement of the C-terminal PDZ domain.

In conclusion, the CDR-induced inhibition of the HTRA2 protease activity seems to be neuroprotective for RGCs during glaucomatous-like conditions in vitro. Therefore, the targeted sequential manipulation of the original CDR peptide (e.g., targeted T(5) to A(5) mutation) could be a promising approach to strengthen the neuroprotective effects towards RGCs in the glaucoma model. However, previous studies have also reported the amelioration effects of HTRA2 protease inhibition by UCF-101 in predominantly proinflammatory diseases such as heart dysfunction [36] and colitis [37]. In contrast with the peptide ligands, the drug compound UCF-101 was occupied directly the catalytic domain of HTRA2 and completely suppressed its proteolytic activity [9], and was also confirmed in the protease activity assay (see Figure 1A–C). Nevertheless, complete inhibition of the proteolytic activity might also result in undesirable side effects, as proper HTRA2 protease function is also needed for neuronal cell survival [10,33]. In addition, it was also reported that UCF-101 induced cell responses independently of its known target HTRA2, and should be considered with caution as a potential drug candidate in the future [38]. Therefore, Zurawa-Janicka et al. (2010) [39] have already postulated the specific regulation of the HTRA2 protease activity via binding of allosteric peptide ligands to the PDZ domain and to carefully adjust the specific proteolytic activity according to the respective type of disease.

For the second part of the present study, we were interested in the identification of direct protein interaction partners of HTRA2 in the retinal tissues of house swine (*Sus scrofa*) and to evaluate the influence of the inhibitor UCF-101 or the synthetic CDR peptide on the HTRA2-specific interactome. Performing co-immunoprecipitation experiments in combination with quantitative MS, we identified in total 50 potential HTRA2-specific retinal interactors in all three groups (see Figure 2 and Table 1) and also validated some of them by targeted MS (see Figure S3). First of all, the majority of the proteins were identified for the first time as potential retinal interaction partners of HTRA2, but, in particular, tubulins (TUBA1C and TUBB, see Table 1) were already verified as HTRA2-specific substrates [40]. Most of the significant binders (33 proteins) were exclusively enriched in the HTRA2 group, whereas up to 76% of these interactions were diminished by additional treatment with 10 µg UCF-101 or 10 µg of synthetic CDR peptide (see Table 1). Particularly interesting representatives of these interactors are the proteins AP3D1 and NCKAP1 (see Figure 3A). Significant interactor AP3D1, for instance, represents a subunit of the adapter protein 3 (AP3) complex, which is involved in lysosomal protein trafficking [41] and also represents an important component for the synaptic vesicle transport in neuronal tissues [42]. Furthermore, the AP3 complex is essential for the removal of cytotoxic α-synuclein deposits in *C. elegans* and triggers neuroprotection via the lysosomal degradation pathway [43,44]. Consistent with this, mutations in the *AP3D1* gene cause serve neurological disorders (e.g., Hermansky−Pudlak syndrome), including immunodeficiency, as well as albinism [45], and can also lead to altered retinal cell differentiation, particularly of amacrine cells, in mice [46]. Another interesting interaction partner NCKAP1 is involved in the important processing of the amyloid-β precursor protein (APP), and was found to be strongly decreased in patients with sporadic Alzheimer’s disease (AD) [47,48]. Disturbed APP processing can result in neurotoxic amyloid plaque formation, which is one of the main hallmarks in AD [49] and was also observed in a chronic glaucoma animal model [50]. In addition, in the microglia of amyotrophic lateral sclerosis (ALS) patients, the decreased expression of NCKAP1 was also significantly correlated with defective phagocytic function and abnormal actin polymerization [51]. However, it can be assumed that both proteins AP3D1 and NCKAP1 are potential substrate molecules of HTRA2 binding to the catalytic domain, as enrichment was clearly inhibited in the UCF-101 or the CDR group (see Figure 3A and Figure S3). Moreover, both interactors induce important regulatory functions by degradation of misfolded protein aggregates preferentially via the endo-lysosomal pathway. In accordance, most of the other significant interactors also represent important components of the ER to Golgi anterograde transport (e.g., ANK2), the protein localization to membrane (e.g., FLNA), and aggrephagy (e.g., PSMC1), and seem to be an important mechanism in the HTRA2-mediated protein network (see Figure 4 and Figure 5A,B). All three binders, ANK2, FLNA, and PSMC1, are also related to amyloid plaque removal by different modes of action (e.g., ubiquitin-mediated protein degradation by PSMC1), and are indispensable for neuronal homeostasis and cell survival [52,53,54]. Interestingly, chaperone molecules (e.g., 14-3-3 or HSP90) play essential roles in the clearance of misfolded proteins by aggrephagy [55,56] and were also found to be up-regulated in the stressed retinal explants [16]. Considering the beneficial effects of the CDR-induced HTRA2 protease inhibition on RGCs ex vivo [16], this might also indicate a pathogenic role of these processes in the pathogenesis of glaucoma; i.a. by hyperactivation. In accordance, overactivation of the HTRA2 proteolytic function has already been reported in AD [14,15] as well as other neurological diseases [33], and is known to trigger autophagy [12]. The role of autophagy in the pathogenesis of glaucoma is still controversial and has so far been associated with RGC survival as well as death [57,58]. Therefore, the specific modulation of the HTRA2 protease activity by specific peptide ligands could be a promising therapeutic strategy in neurodegenerative diseases.

On the contrary, the affinity purification experiments also revealed HTRA2-specific interaction partners, such as CDC42 and SNX3, which were exclusively enriched in the UCF-101 and the CDR group (see Figure 3B and Table 1). Thereby, it can be assumed that the drug compound UCF-101 or the synthetic CDR peptide led to specific conformational changes of HTRA2, facilitating the binding of new interactors. The significant binder CDC42 belongs to the Rho family of GTPases, which are important regulatory molecules maintaining the organization of actin and the microtubule cytoskeleton [59]. CDC42 has drawn attention as an important regulator of neuronal morphology by promoting neurite outgrowth and growth cone development [60,61]. Given the fact, because CDC42 seems to be required for normal retina development and retinal cell survival in the mouse [62], as well as in the zebrafish [63], it might also play an essential role in RGC neuroprotection. With respect to glaucoma, it was shown that CDC42 is responsible for maintaining the tight junction permeability in the trabecular meshwork and regulates its outflow resistance by cytoskeletal rearrangement [64,65]. Increased aqueous humor outflow resistance results in an elevated intraocular pressure (IOP), which is a main risk factor for developing glaucoma [66]. Accordingly, Rho-Kinase (ROCK) inhibitors are attractive treatment options in glaucoma therapy because of their multifunctional IOP-lowering effects [67,68], and might indicate the regulatory function of HTRA2 in this important pathway mechanism. The second interactor, SNX3, is a subunit of the multi-protein complex retromer, which recycles protein cargo from the endosomes to the *trans*-Golgi network [69]. Beyond that, the proper protein function of SNX3 is indispensable for the neuronal development und neuronal function in *C. elegans* [70], and also influences the internalization of APP in vitro [71]. Nevertheless, how exactly these specific interactors contribute to the neuroprotective effects on RGCs ex vivo has to be determined in future studies.

As final result, the MCODE algorithm of the Metascape analysis identified a significant protein interaction network comprising the six HTRA2-specific binders POLR2E, TUBB8, SHMT2, EIF2S3, ME2, CS, and PPA1 (see Figure 5C,D and Table 1). Thereby, this protein network was densely connected with the signaling pathways aerobic respiration and carbon metabolism (see File S3), with SHMT2 as the master regulator. The HTRA2-specific binder SHMT2 is involved in the mitochondrial one-carbon metabolism (1C), which is essential for maintaining the cellular respiration and is also needed for nucleotide biosynthesis [72,73]. Interestingly, SHMT2 is required for the proper assembly of the complex I in the respiratory chain, and seems to represent a novel regulatory link to the 1C metabolism [73]. On the one hand, an increased expression of the active metabolic enzyme SHMT2 is associated with various types of cancer [74,75]. On the other hand, overexpression of SHMT2 is reported to induce neurodegeneration driven by excessive cerebral glycine production [76], and mutations in the *SHMT2* gene are known to cause brain and cardiac developmental disorders [77]. In correlation, transgenic *htra2^mnd^*^2^ mice with a deficient HTRA2 activity also showed an inefficient mitochondrial respiration accompanied by ATP depletion [78], and might reflect the important interaction between SHMT2 and HTRA2 for the proper regulation of the cellular energy metabolism.

## 5. Conclusions

The present study provides, for the first time, a comprehensive protein catalogue of potential interaction partners of the mitochondrial serine protease HTRA2 in retinal tissues of the house swine. Moreover, the co-enrichment of several interaction partners of HTRA2 was diminished by protease inhibitor UCF-101 and/or the synthetic CDR peptide, or elicited binding sites for new interactors. Thereby, the drug compound UCF-101 directly binds to the catalytic domain of HTRA2, blocking its proteolytic activity. The synthetic CDR peptide, in contrast, seems to interact with the C-terminal PDZ domain resulting in hindered substrate accessibility due to specific conformational changes in the catalytic domain. Nevertheless, the molecular interaction with the HTRA2-PDZ domain has to be verified in future studies by using specific protein interaction kinetics or molecular dynamic simulations. However, the majority of the protein interaction partners were associated with interesting pathway mechanisms such as ER to Golgi anterograde transport, the protein localization to membrane, aggrephagy, and pyruvate metabolism/citric acid (TCA) cycle, and illustrate the complexity of the HTRA2-specific interactome. However, how exactly these specific HTRA2-mediated proteins networks are associated with the neuroprotective effects on RGCs ex vivo has be determined in future studies.

## Figures and Tables

**Table 1:** Significant protein interaction partner of the serine protease HTRA2 identified in the HTRA2 group, the HTRA2 + UCF-101 group, and the HTRA2 + CDR group.

No.	Major Protein ID	Protein Name	Gene Name	Score	Peptides	Unique Peptides	Sequence Coverage [%]	Mol. Weight [kDa]	HTRA2	HTRA2 + UCF-101	HTRA2 + CDR
**1**	Q01484	Ankyrin-2	*ANK2*	48.2	14	14	4.7	433.7	√	-	-
**2**	O95782	AP-2 complex subunit alpha-1	*AP2A1*	18.1	8	4	8.9	107.5	√	-	-
**3**	O14617	AP-3 complex subunit delta-1	*AP3D1*	47.0	12	12	13.9	130.2	√	-	-
**4**	P00889	Citrate synthase, mitochondrial	*CS*	6.4	3	3	7.8	51.6	√	-	-
**5**	Q96EY1	DnaJ homolog subfamily A member 3, mitochondrial	*DNAJA3*	22.6	3	3	6	52.5	√	-	-
**6**	Q8TEA8	D-aminoacyl-tRNA deacylase 1	*DTD1*	4.6	1	1	7.2	23.4	√	-	-
**7**	P41091	Eukaryotic translation initiation factor 2 subunit 3	*EIF2S3*	6.4	4	4	13.3	51.1	√	-	-
**8**	Q9NZY2	Putative uncharacterized protein FAM30A	*FAM30A*	3.6	1	1	10.4	14.6	√	-	-
**9**	P21333	Filamin-A	*FLNA*	10.8	3	3	1.7	280.7	√	-	-
**10**	P36915	Guanine nucleotide-binding protein-like 1	*GNL1*	25.8	6	6	11.2	68.7	√	-	-
**11**	Q16775	Hydroxyacylglutathione hydrolase, mitochondrial	*HAGH*	3.6	2	2	6.5	33.8	√	-	-
**12**	Q9Y4L1	Hypoxia up-regulated protein 1	*HYOU1*	24.9	6	6	7.9	111.3	√	-	-
**13**	P23368	NAD-dependent malic enzyme, mitochondrial	*ME2*	7.2	3	3	6.5	65.4	√	-	-
**14**	Q9Y2A7	Nck-associated protein 1	*NCKAP1*	33.0	13	13	12.7	128.8	√	-	-
**15**	Q6VY07	Phosphofurin acidic cluster sorting protein 1	*PACS1*	8.3	2	2	2.5	104.9	√	-	-
**16**	Q01082	Spectrin beta chain, non-erythrocytic 1	*SPTBN1*	38.1	9	9	5	274.6	√	-	-
**17**	Q6S8J3	POTE ankyrin domain family member E	*POTEE*	3.6	9	1	10.4	121.4	√	-	-
**18**	P62191	26S proteasome regulatory subunit 4	*PSMC1*	3.0	1	1	2.7	49.2	√	n.d.	-
**19**	P46405	40S ribosomal protein S12	*RPS12*	7.1	5	5	49.2	14.5	√	n.d.	-
**20**	P55735	Protein SEC13 homolog	*SEC13*	4.5	2	2	8.1	35.5	√	n.d.	n.d.
**21**	P11493	Serine/threonine-protein phosphatase 2A catalytic subunit beta isoform (Fragment)	*PPP2CB*	3.8	1	1	3.8	33.6	√	n.d.	n.d.
**22**	Q92777	Synapsin-2	*SYN2*	9.3	6	4	12	63.0	√	n.d.	n.d.
**23**	P34897	Serine hydroxymethyltransferase, mitochondrial	*SHMT2*	3.6	2	2	4.4	56.0	√	-	n.d.
**24**	P13984	General transcription factor IIF subunit 2	*GTF2F2*	9.7	4	4	20.1	28.4	-	√	-
**25**	Q95250	Membrane-associated progesterone receptor component 1	*PGRMC1*	10.8	2	2	17	21.6	-	√	-
**26**	Q15181	Inorganic pyrophosphatase	*PPA1*	47.5	5	5	21.1	32.7	-	√	-
**27**	O14579	Coatomer subunit epsilon	*COPE*	3.5	1	1	5.5	34.5	n.d.	√	-
**28**	Q99747	Gamma-soluble NSF attachment protein	*NAPG*	22.6	5	5	18.9	34.7	-	-	√
**29**	O43660	Pleiotropic regulator 1	*PLRG1*	3.9	4	4	11.1	57.2	-	-	√
**30**	P19388	DNA-directed RNA polymerases I, II, and III subunit RPABC1	*POLR2E*	21.5	2	2	12.4	24.6	-	-	√
**31**	Q8N474	Secreted frizzled-related protein 1	*SFRP1*	10.2	3	3	14	35.4	-	-	√
**32**	Q9BQE3	Tubulin alpha-1C chain	*TUBA1C*	2.6	22	1	64.6	49.9	-	-	√
**33**	Q5T653	39S ribosomal protein L2, mitochondrial	*MRPL2*	4.5	1	1	8.2	33.3	n.d.	-	√
**34**	Q2M3V2	Ankyrin repeat domain-containing protein SOWAHA	*SOWAHA*	2.5	1	1	1.5	57.4	n.d.	-	√
**35**	Q3ZCM7	Tubulin beta-8 chain	*TUBB8*	14.5	11	2	28.2	49.8	n.d.	-	√
**36**	**O43464**	**Serine protease HTRA2, mitochondrial**	***HTRA2***	**323.3**	**18**	**18**	**52.8**	**48.8**	**√**	**√**	**√**
**37**	Q15185	Prostaglandin E synthase 3	*PTGES3*	4.3	2	2	15.6	18.7	√	√	√
**38**	Q13404	Ubiquitin-conjugating enzyme E2 variant 1	*UBE2V1*	3.0	2	1	12.9	16.5	√	√	√
**39**	P09038	Fibroblast growth factor 2	*FGF2*	5.4	3	3	9.7	30.8	-	√	√
**40**	P19130	Ferritin heavy chain	*FTH1*	10.6	3	2	21.5	21.0	-	√	√
**41**	Q6QA76	PDZ domain-containing protein 11	*PDZD11*	11.6	3	3	35.7	16.2	-	√	√
**42**	Q007T2	Cell division control protein 42 homolog	*CDC42*	19.3	4	4	22	21.3	n.d.	√	√
**43**	O60493	Sorting nexin-3	*SNX3*	5.0	3	2	18.5	18.8	n.d.	√	√
**44**	Q9Y5L4	Mitochondrial import inner membrane translocase subunit Tim13	*TIMM13*	7.1	1	1	14.7	10.5	n.d.	√	√
**45**	P61088	Ubiquitin-conjugating enzyme E2 N	*UBE2N*	3.3	2	2	16.4	17.1	-	√	√
**46**	Q13825	Methylglutaconyl-CoA hydratase, mitochondrial	*AUH*	8.2	5	5	19.2	35.6	√	-	√
**47**	Q29290	Cystatin-B	*CSTB*	2.5	1	1	12.2	11.1	√	-	√
**48**	P31150	Rab GDP dissociation inhibitor alpha	*GDI1*	4.7	3	2	11.4	50.6	√	-	√
**49**	Q04760	Lactoylglutathione lyase	*GLO1*	3.3	2	2	12.5	20.8	√	-	√
**50**	O00116	Alkyldihydroxyacetonephosphate synthase, peroxisomal	*AGPS*	10.3	1	1	2.6	72.9	√	n.d.	√
**51**	Q9ULC4	Malignant T-cell-amplified sequence 1	*MCTS1*	4.5	1	1	10.5	20.6	√	n.d.	√

**√:** Proteins fulfilled filtering criteria (*p* < 0.01; log_2_ fold change > 1.5); **-:** Proteins failed to fulfill the filtering criteria (*p* > 0.01; log_2_ fold change < 1.5); **n.d.:** Proteins were not detectable in at least three biological replicates of the respective sample group (HTRA2, UCF-101, or CDR groups); **bold:** Target protein HTRA2.

## Data Availability

MS data are available via ProteomeXchange with identifier PXD024442.

## Supplementary Materials

The following are available online at https://www.mdpi.com/article/10.3390/biomedicines9081013/s1. The supplementary material consists of supplementary Files S1–S3 and supplementary Figures S1–S3. File S1 contains the protein identifications of the mass spectrometric (MS) analysis and File S2 contains the statistical analysis of the MS output data. File S3 contains the results of the pathway analyses using bioinformatics tool DAVID and functional annotation tool Metascape. Figure S1 indicates the mutational analysis of the potential CDR binding motif. Figure S2 shows the 1-D SDS PAGE of the eluate fractions (50 µg per lane) of the different magnetic bead groups (CTRL, HTRA2, UCF-101, and CDR group). Figure S3 illustrates the verification of HTRA2-specific interaction partners by targeted MS.
