# Supplementary figures and images for: Regulation of the HTRA2 Protease Activity by an Inhibitory Antibody-Derived Peptide Ligand and the Influence on HTRA2-Specific Protein Interaction Networks in Retinal Tissues
Source: Biomedicines. 2021 Aug 13;9(8):1013. doi: 10.3390/biomedicines9081013 (PMC8427973; doi:10.3390/biomedicines9081013)

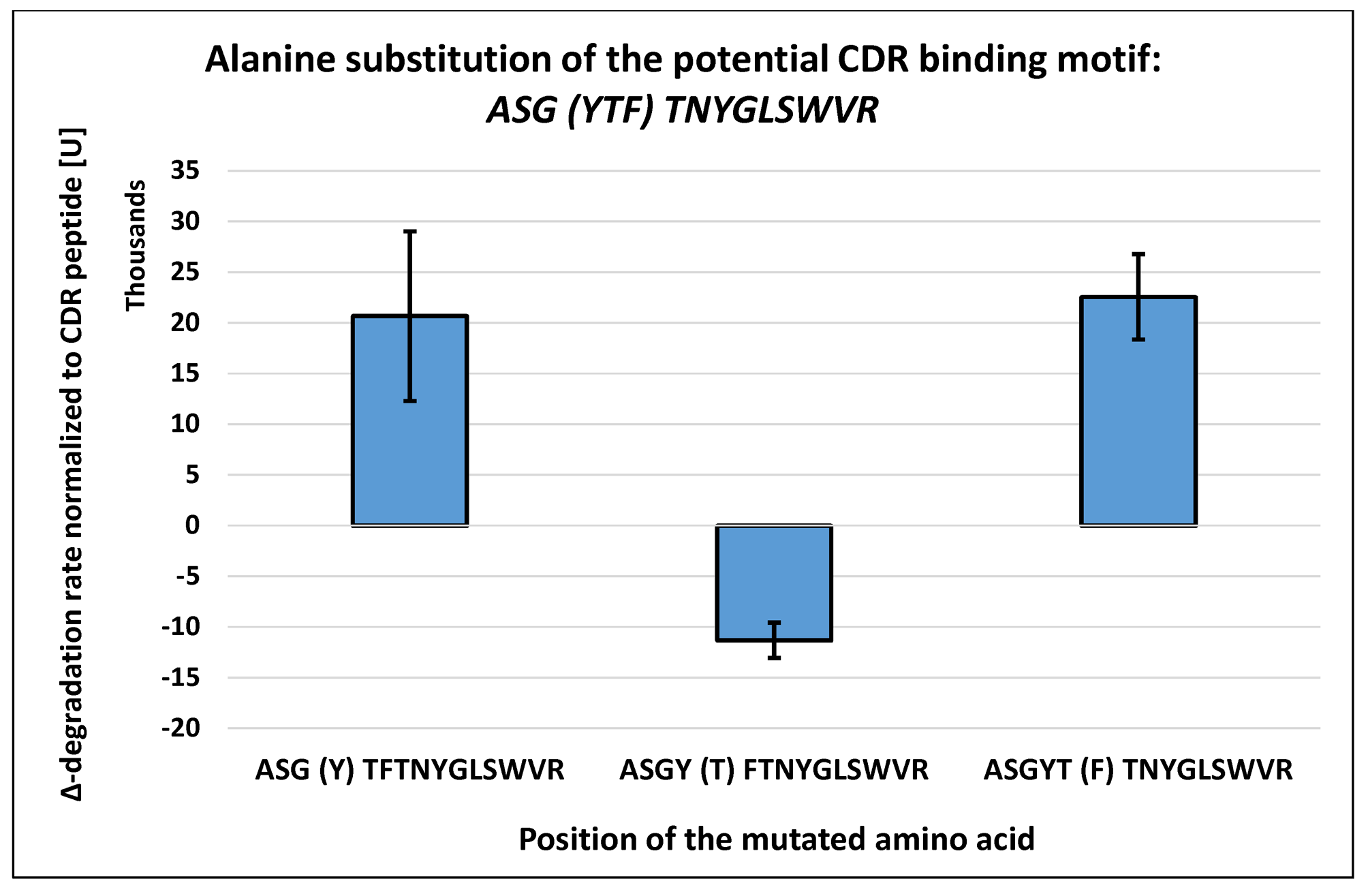

Supplement: Supplementary file 1 [file biomedicines-09-01013-s001.zip › biomedicines-1292337-supplementary/Supplementary Figure S1.tif]

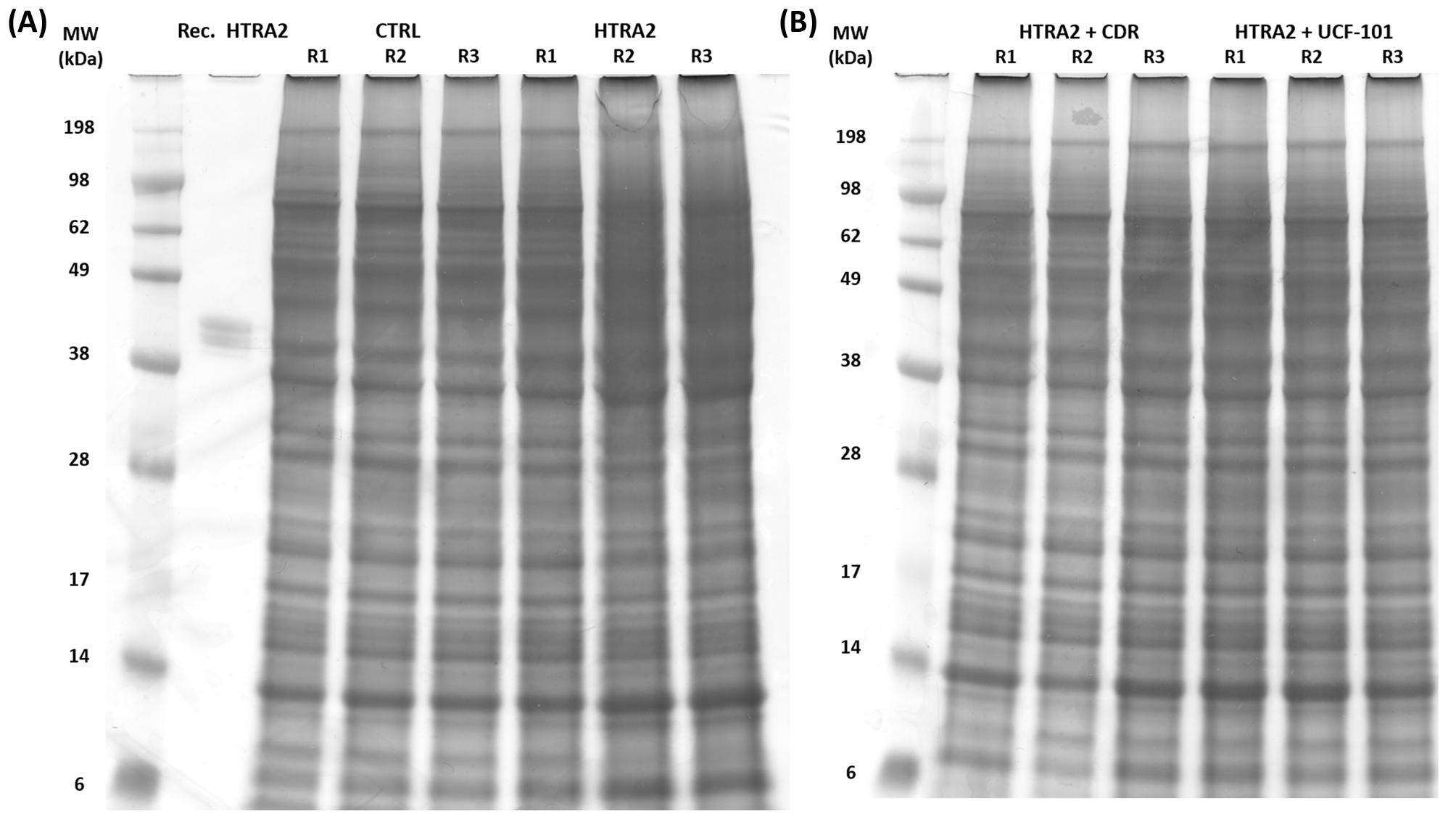

Supplement: Supplementary file 1 [file biomedicines-09-01013-s001.zip › biomedicines-1292337-supplementary/Supplementary Figure S2.tif]

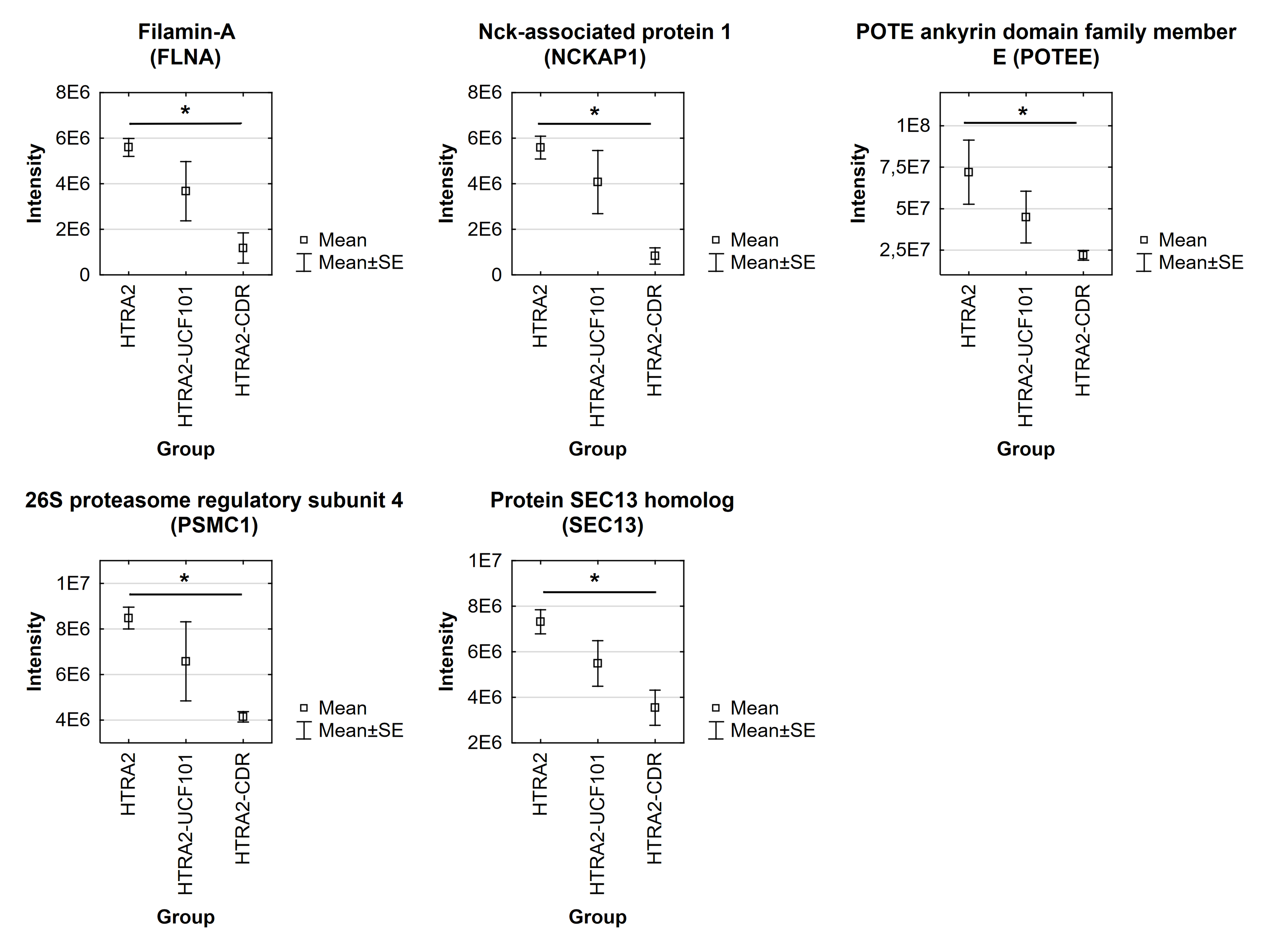

Supplement: Supplementary file 1 [file biomedicines-09-01013-s001.zip › biomedicines-1292337-supplementary/Supplementary Figure S3.tif]
